# An Epidemiological Study on COVID-19: A Rapidly Spreading Disease

**DOI:** 10.7759/cureus.7313

**Published:** 2020-03-18

**Authors:** Hussein H Khachfe, Mohamad Chahrour, Julie Sammouri, Hamza Salhab, Bassel Eldeen Makki, Mohamad Fares

**Affiliations:** 1 General Surgery, American University of Beirut Medical Center, Beirut, LBN; 2 Surgery, American University of Beirut Medical Center, Beirut, LBN; 3 Medicine, American University of Beirut Medical Center, Beirut, LBN; 4 Medicine, Tehran University of Medical Sciences, Tehran, IRN; 5 Sports Medicine, American University of Beirut Medical Center, Beirut, LBN

**Keywords:** coronavirus, novel coronavirus, covid-19, infectious disease, epidemiology

## Abstract

Background

The outbreak of the novel coronavirus disease in 2019 (COVID-19) caused a major public health crisis worldwide and challenged healthcare systems across the six continents. The high infectivity of the disease led many governments to adopt strict regulations and measures with the aim of containing its spread. The purpose of this study is to assess the incidence, severity, and territorial expansion of COVID-19.

Methods

Data from the World Health Organization was screened, and COVID-19 situation reports were extracted from January 21 up till March 14 (inclusive). Our data included the total number of cases, total number of new cases, total number of cured cases, and total number of related deaths. Percentage change of cases over the days of our study were calculated using the Joinpoint regression, with a significance level set at greater than 0.05.

Results

The total number of COVID-19 cases reached 156,622, with 5,845 subsequent deaths. China, Italy, and Iran have the highest number of cases worldwide. During the first 22 days, the incidence rate of COVID-19 increased significantly to reach 1.81 cases per million persons (p<0.001). That was followed by a significant decrease over the next 11 days (p<0.001) to reach 0.071 cases per million persons. A steady rise then followed, which saw a significant increase in incidence rate to 1.429 cases per million persons (p<0.001). Percentages of death and cured cases varied across the different countries; nevertheless, death percentages have generally been decreasing since the start of the crisis.

Conclusion

Adopting precautionary regulations such as social isolation, increasing sanitation, and employing strict quarantine measures have proved to be beneficial in containing the virus. Further research needs to be conducted to help discover therapeutic modalities and improve outcomes.

## Introduction

In December 2019, the capital of the Chinese province Hubei, Wuhan city, witnessed an outbreak of “pneumonia of unknown source” attributed to a newly identified culprit: a novel coronavirus [1]. The clinical findings among most patients were dry cough, dyspnea, and fever. This led the Centers for Disease Control and Prevention (CDC) to designate the pathogen as “severe acute respiratory syndrome coronavirus 2” or SARS-CoV-2 before the World Health Organization (WHO) termed the disease itself COVID-19 (coronavirus disease in 2019) in January 2020 [2]. This epidemic is the third coronavirus outbreak in the last 20 years after the SARS-CoV and the Middle East respiratory syndrome MERS-CoV [3].

Initially, the outbreak seemed to have been caused by zoonotic transmission in the setting of a seafood market in Wuhan, where wild animals were sold. It was a possibility that the animals could have served as disease reservoirs, but that was not confirmed by any credible source [4]. It was not long before droplet and contact person-to-person transmission became known as the primary mode of transmission [5]. This led to a rapid increase in the number of cases in China outside Wuhan, with 31.3% of all patients having recently visited the city and 72.3% having recently been in contact with its residents [6]. On January 30, 2020, the WHO announced a state of international public health emergency, later declaring COVID-19 to be a pandemic in March 2020 [7]. Despite a significant infectivity rate, the case-fatality rate among confirmed cases of COVID-19 is estimated at 3.7%, with the majority of deaths typically occurring among the elderly (age > 80 years), patients having multiple comorbidities, and the immunocompromised population [8].

International directives issued by the WHO and the CDC are limited to supportive treatment and prevention to achieve infection control [9]. For the time being, there are no vaccines or antiviral treatments for the coronavirus family. This has led several researchers throughout the world to investigate the efficacy of drugs such as interferon-alpha and lopinavir (previously used to treat SARS) along with other novel pharmacological therapies [9].

In this study, our aim is to assess the incidence, severity, and territorial expansion of COVID-19 and give recommendations on how to limit the spread of the disease.

## Materials and methods

Case data extraction

Data from the WHO Health Emergency Dashboard novel COVID-19 situation reports were extracted, from its initiation (January 21, 2020) up until the most recent one (March 14, 2020) [10]. These data were stratified into the total number of cases, total number of new cases, total number of cured cases, and total number of deaths. Each of these categories was classified per country.

The percentage of deaths and patients cured were also extracted from the aforementioned database. These were used to determine the efficacy of the government and healthcare systems of the affected countries.

The percentage change of cases over the days of our study was calculated using the Joinpoint regression model. Statistical analysis was performed using Joinpoint 4.7.0.0, with a significance level set at greater than 0.05.

## Results

Overall

**Figure 1:**
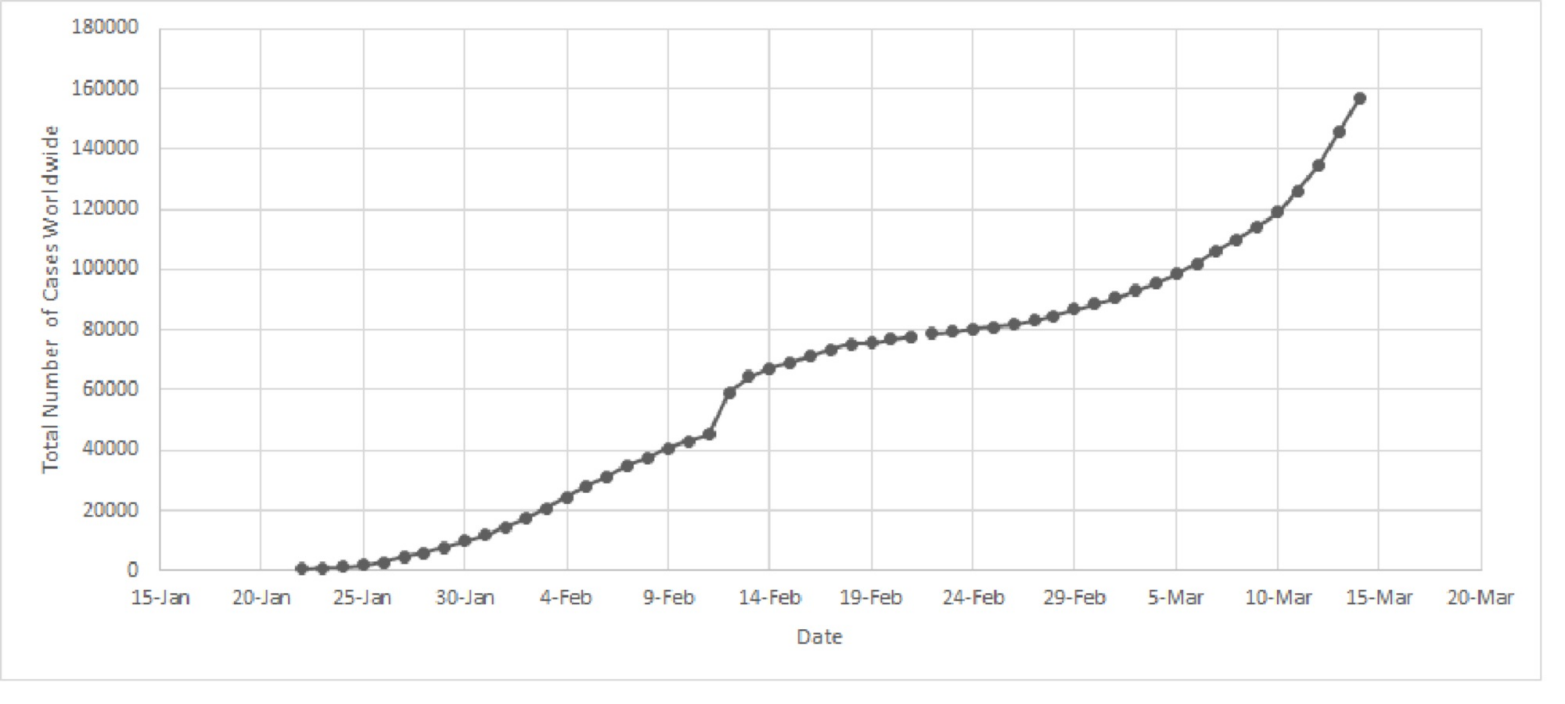
Increase in the total number of novel coronavirus disease 2019 (COVID-19) from January 22 to March 14, 2020.

**Figure 2:**
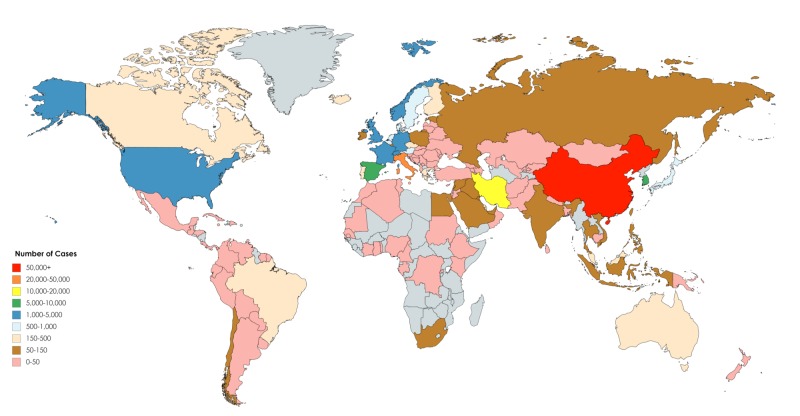
Worldwide distribution of the novel coronavirus disease 2019 (COVID-19) as of March 14, 2020.

The total number of COVID-19 cases as of March 14, 2020, is 156,622, spread across 154 countries worldwide (Figure 1). China, Italy, and Iran are the countries with the highest number of cases worldwide, with a total of 80,849, 21,157, and 12,729 cases, respectively (Table 1, Figure 2).

**Table 1:** Distribution of the total number of cases, cured cases, and deaths in the top 20 countries impacted by the novel coronavirus disease 2019 (COVID-19) as of March 14, 2020.

Country	Total Cases	Total Deaths	Total Cured
China	80,849	3,199	66,916
Italy	21,157	1,441	1,966
Iran	12,729	611	4,339
S. Korea	8,162	75	834
Spain	6,391	196	517
Germany	4,649	9	46
France	4,499	91	12
USA	3,045	60	56
Switzerland	1,375	13	4
UK	1,140	21	18
Norway	1,126	3	1
Sweden	961	2	1
The Netherlands	959	12	2
Denmark	836	1	1
Japan	825	22	144
Austria	800	1	6
Belgium	689	4	1
Qatar	337		4
Australia	283	5	27

A total of 5,845 deaths from 47 different countries have been registered from COVID-19 as of March 14, 2020. The country most affected was China with 3,199 deaths followed by Italy with 1,411 deaths and Iran with 611 deaths (Table 1). A total of 75,943 patients have recovered worldwide from COVID-19 as of March 14, 2020. The country with the most resolved cases was China with 66,916 followed by Iran with 4,339 cases and Italy with 1,966 (Table 1).

Trends of new cases

**Figure 3:**
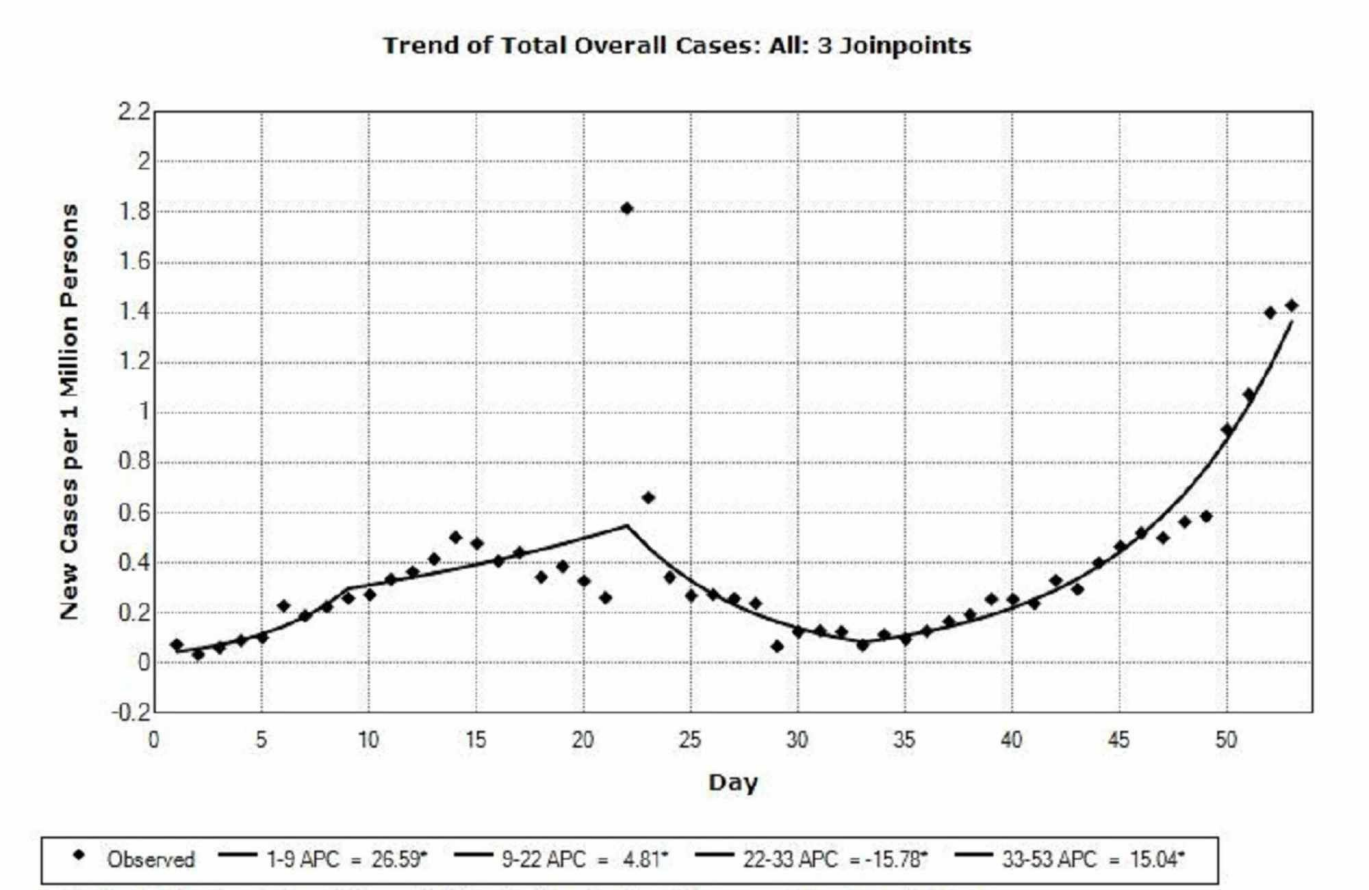
Trends and percentage change of new cases worldwide from the novel coronavirus disease 2019 (COVID-19) as of March 14, 2020.

As per Figure 3, our analysis shows a significant increase in the number of new COVID-19 cases worldwide from 0.074 cases per million persons at day 1 (January 22) to 1.81 cases per million persons at day 22 (February 13) (p<0.001). A significant decrease then occurs between day 22 (February 13) and day 33 (February 24), from 1.81 cases per million persons to 0.071 cases per million persons (p<0.001). This is then followed by a significant increase that occurs as of day 33 (February 24) and up until day 53 (March 14), from 0.071 cases per million persons to 1.429 cases per million persons (p<0.001).

Cured and death percentage of resolved cases from the top three affected countries

**Figure 4:**
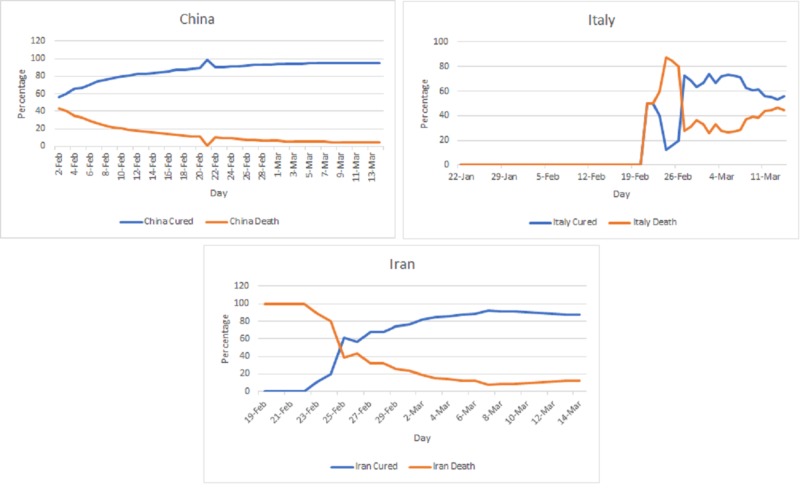
Outcomes of resolved cases (percentage of deaths and cured cases) from the novel coronavirus disease 2019 (COVID-19) as of March 14, 2020.

The cured and death percentages of the top three affected countries, i.e., China, Italy, and Iran, can be seen in Figure 4. The cured percentage in China continuously increased from 56.82% on February 2, 2020, to 95.44% on March 14, 2020. The death percentage among the resolved cases has continuously decreased from 43.18% to 4.56%.

In Italy, the percentage of deaths among resolved cases continuously increased to reach a maximum value of 84.62% on February 25, 2020. The percentage of deaths then decreased temporarily to reach 38.59% on March 10 before increasing again to reach a value of 44.3% on March 14, 2020.

In Iran, the percentage of deaths among resolved cases was 100% between February 19, 2020, and February 22, 2020. This percentage started to drop continuously to a minimum of 12.34% on March 14 coupled with a rise in the percentage of cured cases that reached 87.66%.

## Discussion

As described in the Results section, there have been a total of 156,622 confirmed cases of COVID-19 spread across 154 countries worldwide. These cases occurred over the course of 53 days and spanned over six continents through person-to-person transmission, highlighting an extremely high infectivity rate [11].

The 5,845 deaths caused by COVID-19 as of March 14, 2020, equate to around 3.7% of all infected cases. This is relatively better than the rates of previous SARS-CoV and MERS-CoV, which reached 10% and 35%, respectively [8]. This is also complemented by a high recovery rate, where more than 75,000 patients fully recovered from the disease. Nevertheless, precautionary measures remain an obligation, and prevention protocols need to be set, regardless of the relatively favorable prognosis of this disease, when compared with its predecessors.

The initial 22 days of reporting showed a significant increase in the overall disease cases worldwide. This was explained by the continuous and chain-like human-to-human transmission at the birth site of the disease in China, along with poor restrictions on travel and transit [12]. This led to a rapid and prevalent spread that reached many nearby regions and countries. However, a significant decrease in incidence followed during the next 11 days, mainly due to decisions taken by the Chinese government and the Chinese CDC [13]. These decisions ranged from raising awareness, disinfection, and declaring states of emergency, to building make-shift hospitals for patient management [3,14]. This helped contain the disease in China and led to an eventual decrease in incidence. Nevertheless, a subsequent significant increase in worldwide incidence ensued as of day 33 onward due to the rapid spread of the disease across the Chinese borders. Many countries did not possess the required resources and facilities to face such a pandemic and were, thus, vulnerable to its high infectivity.

The main countries affected outside of China are Italy in Europe and Iran in Asia, where cases continue to increase at an alarming rate. What is even more concerning is that the percentage of deaths from resolved cases in the latter countries is prominently high. In Italy, the death rate fluctuated at the beginning, increasing and then decreasing, before witnessing a steady rise that has been going on up until day 53. It is evident that the Italian healthcare system was taken by surprise with respect to the viral outbreak, and this lack of preparation explains the initial increase in death rates [15]. The subsequent decrease can be explained by the initial attempts at containment proposed by the Italian government; however, oversaturation of the hospitals and facilities caused a subsequent rise in the death rate that has been going on steadily [16]. On the other hand, Iran had a mortality rate of 100% during the first couple of days of reporting. This can be warranted as under-reporting from the Iranian government due to lack of screening resources possessed at the time as well as the vulnerable healthcare system present, which could not properly contain or adequately treat the disease in the affected populations [17]. Subsequent continuous decrease in mortality rates coupled with increases in recovery rates followed up until the most recent reports.

COVID-19 has become a worldwide pandemic affecting all inhabited continents of Earth. Although Asia was the epicenter of the disease during the start of the COVID-19 pandemic with the major outbreaks in China, Iran, South Korea, and Japan, other continents must not be forgotten. With the containment that occurred in China and the decreasing death rates in Iran, attention has shifted to European countries exhibiting increasing incidence such as Germany, France, and Switzerland. The significance of fighting COVID-19 more aggressively in Europe revolves around the old-age structure found in its countries, which means that there are more vulnerable people affected [18]. Africa, on the other hand, suffers from abysmal medical infrastructure and would require international aid to help stop preventable deaths caused by the disease [19]. Parallels can be drawn to South America, which only further proves that this pandemic will not end except with a collective global effort.

We must adhere to infectious control guidelines in order to help contain the disease and decrease the number of people being hospitalized. People exposed to the virus and showing symptoms must undergo polymerase chain reaction (PCR) test and, if positive, be isolated and treated. If the test turns out negative, it is advised to remain in home quarantine for 14 days. People who are exposed to the virus but do not show any symptoms must remain in home quarantine for 14 days. People who exhibit symptoms but have not been exposed to the disease must check with a medical doctor, implement safety precautions (proper coughing and sneezing etiquette), and avoid going out unless necessary. Finally, those who show no symptoms and have not been exposed to the disease are recommended to practice continuous hand hygiene and adhere to precautionary measures (washing hands with soap for at least 20 seconds, use of antiseptics, avoid touching the face, etc.). Avoiding unnecessary social interactions is also essential for the prevention of exposure.

The lessons that can be learnt from the Chinese model are that good isolation, quarantine, and sanitization are the best measures against this global disease. Although this virus has affected many countries and populations, there is still a lot to be known regarding its pathogenicity and viral characteristics. More research and studies must be conducted to help fight off this virus, discover possible vaccines or cures, and establish safety plans for future epidemics or pandemics [20-22].

## Conclusions

COVID-19 has impacted the world in a way that could not have been predicted. Its rapid expansion over six continents, and in such a short period of time, delineates its high infective potential. The high mortality rates observed in some nations served to show how unprepared our healthcare systems were when facing such a global pandemic. Nevertheless, the healthcare plans adopted by China have proved that social distancing, increasing sanitation, and employing full quarantine can be very beneficial for containing this pandemic. As the worldwide incidence rate increases, governments are urged to take strict measures and enforce stringent precautions to help contain the disease, limit its spread, and decrease its subsequent mortality rate.

## References

[1] Purcell LN, Charles AG (2020). An Invited Commentary on “World Health Organization declares global emergency: A review of the 2019 novel Coronavirus (COVID- 19)": Emergency or new reality?. Int J Surg.

[2] Zhou F, Yu T, Du R (2020). Clinical course and risk factors for mortality of adult inpatients with COVID-19 in Wuhan, China: a retrospective cohort study. Lancet.

[3] Rothan HA, Byrareddy SN The epidemiology and pathogenesis of coronavirus disease (COVID-19) outbreak. J Autoimmun.

[4] Guo YR, Cao QD, Hong ZS (2020). The origin, transmission and clinical therapies on coronavirus disease 2019 (COVID-19) outbreak - an update on the status. Mil Med Res.

[5] Yang Y, Peng F, Wang R (2020). The deadly coronaviruses: the 2003 SARS pandemic and the 2020 novel coronavirus epidemic in China. J Autoimmun.

[6] Guan WJ, Ni ZY, Hu Y (2020). Clinical characteristics of coronavirus disease 2019 in China. New Engl J Med.

[7] Guo Y, Huang YM, Huang J (2020). COVID-19 pandemic: global epidemiological trends and China's subsequent preparedness and responses. Zhonghua Liu Xing Bing Xue Za Zhi.

[8] Cascella M, Cuomo A, Cuomo A (2020). Features, Evaluation and Treatment Coronavirus (COVID-19). https://www.ncbi.nlm.nih.gov/books/NBK554776/.

[9] Sohrabi C, Alsafi Z, O'Neill N (2020). World Health Organization declares global emergency: A review of the 2019 novel coronavirus (COVID-19). Int J Surg.

[10] (2020). Coronavirus disease (COVID-2019) situation reports. disease (COVID-2019) situation reports. 2020.

[11] Wang Y, Wang Y, Chen Y, Qin Q (2020). Unique epidemiological and clinical features of the emerging 2019 novel coronavirus pneumonia (COVID-19) implicate special control measures. J Med Virol.

[12] Riou J, Althaus CL (2020). Pattern of early human-to-human transmission of Wuhan 2019 novel coronavirus (2019-nCoV), December 2019 to January 2020. Euro Surveill.

[13] Xiong Z, Fu L, Zhou H (2020). Construction and evaluation of a novel diagnosis process for 2019-Corona Virus Disease. Zhonghua Yi Xue Za Zhi.

[14] (2020). Wuhan closes last makeshift coronavirus hospital as China's infection rate falls. https://www.theguardian.com/world/video/2020/mar/10/wuhan-closes-last-makeshift-coronavirus-hospital-video.

[15] Ferre F, de Belvis AG, Valerio L (2014). Italy: health system review. Health Syst Transit.

[16] Remuzzi A, Remuzzi G (2020). COVID-19 and Italy: what next?. Lancet.

[17] (2020). Iran elections: record low turnout but hardliners set for win. https://www.bbc.com/news/world-middle-east-51605942.

[18] Christensen K, Doblhammer G, Rau R, Vaupel JW (2009). Ageing populations: the challenges ahead. Lancet.

[19] Mooketsane KS, Phirinyane MB (2015). Health governance in Sub-Saharan Africa. Glob Soc Policy.

[20] Fares J, Khachfe HH, Fares MY, Salhab HA, Fares Y (2019). Conflict medicine in the Arab world. Handbook of Healthcare in the Arab World.

[21] Fares J, Salhab HA, Fares MY (2019). Academic medicine and the development of future leaders in healthcare. Handbook of Healthcare in the Arab World.

[22] Fares MY, Salhab HA, Khachfe HH, Fares Y, Fares J (2019). Sports medicine in the Arab world. Handbook of Healthcare in the Arab World.

